# A novel defined cuproptosis-related gene signature for predicting the prognosis of colon adenocarcinoma

**DOI:** 10.3389/fonc.2022.927028

**Published:** 2022-11-25

**Authors:** Bixian Luo, Jianwei Lin, Anqi Ni, Wei Cai, Xinbo Yu, Mingliang Wang

**Affiliations:** ^1^ Department of General Surgery, Ruijin Hospital, Shanghai Jiao Tong University School of Medicine, Shanghai, China; ^2^ Kidney Disease Center, The First Affiliated Hospital, Zhejiang University School of Medicine, Institute of Nephrology, Zhejiang University, Key Laboratory of Kidney Disease Prevention and Control Technology, Zhejiang Province, Zhejiang Clinical Research Center of Kidney and Urinary System Disease, Hangzhou, Zhejiang, China; ^3^ Department of Urology, Ruijin Hospital, Shanghai Jiao Tong University School of Medicine, Shanghai, China; ^4^ Department of General Surgery, Ruijin Hospital Luwan Branch, Shanghai Jiao Tong University School of Medicine, Shanghai, China

**Keywords:** cuproptosis, colon adenocarcinoma, risk score, gene signature, nomogram

## Abstract

The prognosis of colon adenocarcinoma (COAD) needs to be improved. Cuproptosis is a recently discovered cell death caused by intracellular overload of copper ions. There have been no reports about the cuproptosis-related prognostic model in COAD. First, we screened 30 differentially expressed genes (DEGs) from patients with COAD using The Cancer Genome Atlas (TCGA) database. Gene Expression Omnibus (GEO) database was used as a validation set to establish a risk model of five cuproptosis-related genes (CKDN2A, SDHB, CCS, ULK1, and CMC1) by least absolute shrinkage and selection operator (LASSO) Cox regression analysis. In both TCGA and GEO cohorts, we could see that overall survival of COAD patients of the low-risk group was longer. Combined with the clinical characteristics, the risk score was found to be an independent prognostic factor. Furthermore, single-sample Gene Set Enrichment Analysis (ssGSEA) showed that the levels of Th1 and Treg immune cells changed both in TCGA and GEO databases. Finally, clinical samples were used to verify the mRNA and protein levels of five risk-model genes. In conclusion, this model could predict the prognosis of COAD patients, and the mechanism may be related to the changes in immune cells in the tumor microenvironment (TME).

## Introduction

Colon cancer is a big threat to public health and the leading cause of cancer-induced death in the world (1). There was an estimated 1.198 million new cases and 0.576 million new deaths of colon cancer worldwide in 2020 (2). In the United States, with an estimated 104,270 new cases and 52,980 new deaths, colon cancer is the third most commonly diagnosed and leading cause of cancer death in 2021 (1, 2). The 5-year relative survival rate for persons with colon cancer is 64%, which suggests that colon cancer has poor prognosis (3). Meanwhile, adenocarcinoma is the main pathological type of colon cancer (4). Therefore, it is necessary to find more biomarkers with clinical application value to predict the prognosis of colon adenocarcinoma (COAD) patients. Obviously, we need to build a reliable novel prognostic model to make it more feasible.

The modes of cell death are trend topics for cancer research due to its effects on tumor cells and microenvironment (5, 6). Cuproptosis is the copper-induced form of regulated cell death, which differs from classical apoptosis, pyroptosis, necroptosis, and ferroptosis (7). Recent research published in Science showed that intracellular copper accumulation induced cell death by binding copper to lipoylated proteins of the tricarboxylic acid (TCA) cycle. This biological mechanism leads to lipoylated proteins aggregation in mitochondria and the decrease in Fe–S cluster proteins (8). The cytoplasm homeostatic state was broken, which contributes to mitochondrial proteotoxic stress and ultimately cell death (8, 9). Thus, cuproptosis-related genes may lead to the inhibition of tumor cells growth, influence immune cells in TME, and serve as the potential biomarkers to predict the prognosis of COAD to provide feasible guidance for clinical application.

In this study, we first wanted to select differentially expressed cuproptosis-related genes between normal and tumor tissues of COAD patients in TCGA database to obtain 30 DEGs. Then, a risk model based on cuproptosis-related genes was constructed from DEGs by univariate and least absolute shrinkage and selection operator (LASSO) Cox regression analysis. Next, a nomogram established by the clinicopathological features and risk model was used to further explore its clinical application of predicting prognosis in COAD patients. Moreover, we found that this risk model might have a relationship with changing subtypes of T-helper immune cells to remodulate TME. Finally, the tumor tissue and their paired normal tissue from 13 patients with COAD were used to verify the gene mRNA and protein expressions in the model.

## Methods

### Data collection of TCGA and GEO databases

Reads per kilobase per million mapped reads (RPKM) data of COAD RNA-seq were downloaded from the TCGA database (https://portal.gdc.cancer.gov/) along with the clinical data of the corresponding samples. Microarray data (GSE39582) from the GEO database (http://www.ncbi.nlm.nih.gov/geo) was used as the validation set, including RNA-seq data and clinical information of COAD patients. The following corresponding clinical characteristics were extracted: age, gender, tumor stage, lymph node stage, and metastasis stage (T stage, N stage, and M stage) based on the American Joint Committee on Cancer.

### Identification of differentially expressed genes

A total of 44 cuproptosis-related genes were extracted from known literature and are provided in **Supplementary Table S1** (7–9). The “limma” package was used to identify DEGs between adjacent normal and tumor tissues in the TCGA-COAD cohort with a p-value <0.05 (10). The heatmap was constructed by “pheatmap” package. The protein–protein interaction (PPI) network of cuproptosis-related DEGs was generated by STRING database (11, 12). The “reshape2” package and “igraph” package were used to construct the correlation network for DEGs.

### Construction of risk score model by univariate Cox and LASSO Cox regression analyses

We first used univariate Cox regression analysis to screen the curoptosis-related genes significantly associated with prognosis of COAD patients. Then, the LASSO Cox regression was performed to establish the risk score model, and the risk score for each COAD patients was calculated. The COAD patients were divided into high- and low-risk group according to the median cutoff value. Kaplan–Meier analysis with log-rank test was used to compare the difference in overall survival (OS) between two subgroups. Principal component analysis (PCA) based on the expression of five risk genes was used to show its efficacy of separate high- and low-risk patients and was performed by “stats” R package. Furthermore, univariate and multivariable Cox regression were employed for evaluating whether risk score could be an independent predictor of OS for COAD patients.

### Construction of the nomogram to evaluate the clinical applications of COAD patients

A nomogram model was constructed using R-package “rms” based on risk score and other clinical factors (age and stage) to forecast the survival rate of 1, 3, and 5 years for COAD patients. To estimate the consistency between actual and predicted survival, calibration charts were also drawn to evaluate its consistency with actual prognosis of COAD patients in TCGA based on nomogram. We used GSE39582 as external validation cohort.

### Functional enrichment analysis

GO was used to show the biological process, cellular component, and molecular function of COAD based on DEGs selected by two different risk groups. KEGG was performed to explore the potential molecular mechanisms and biological functions based on DEGs by using R-package “ClusterProfiler,” the significance threshold was p-value <0.050 (13). The single-sample Gene Set Enrichment Analysis (ssGSEA) and “gsva” R package were used to calculate the infiltrating score of immune cells and the level of immune-related function.

### Tissues of COAD patients

Sixteen pairs of tumor and normal tissues were obtained from COAD patients in the Department of General Surgery, Ruijin Hospital, School of Medicine, Shanghai Jiao Tong University. Fresh samples were frozen in liquid nitrogen and stored at −80°C for mRNA and protein extraction. Patients had given written informed consent for tissue collection and analysis of clinical data. The study was approved by the ethics committee of Ruijin Hospital.

### RT-PCR of human samples

Total RNA was extracted using Total RNA Isolation Reagent (Biosharp, China) and then reverse transcribed into cDNA using PrimeScript™ RT Reagent Kit (Takara, China). The qPCR was performed using TB Green^®^ Premix Ex Taq™ II (Takara, China) on the 7,500 Fast Real-Time PCR System (Applied Biosystems, CA, USA). The 2^−ΔCt^ method was used to calculate gene transcription level, with β-actin mRNA as control. The primer sequences are listed in **Table 1**.

**Table 1:** Primer sequences of genes in the risk model used for qPCR.

Gene	Primer sequence
β-actin	F: GACCTGTACGCCAACACAGT R: CTCAGGAGGAGCAATGATCT
CDKN2A	F: ATGGAGCCTTCGGCTGACTG R: TCATGACCTGGATCGGCCT
SDHB	F: TGAACATCAATGGAGGCAACAC R: GTCCAGTTTCTCACGCTCTTCT
CCS	F: CAGTACGGGGACCTTACAAACA R: GCTGCTCATCCTCCATTCTGA
ULK1	F: TTCCAAACACCTCGGTCCTCT R: AACTTGAGGAGATGGCGTGTAG
CMC1	F: GATGTCTCTTCGTCTACCCTTCC R: GAGATGCTGGTTTGCTCCTGA

### Western blot analysis of human samples

Human samples were dissociated and lysed in radioimmunoprecipitation (RIPA) buffer supplemented with protease inhibitor cocktail (Epizyme Biotech, China). Its concentration was measured with a BCA protein assay kit (Takara Bio, Tokyo, Japan). Protein samples were separated by electrophoresis on sodium dodecyl sulfate–polyacrylamide gel electrophoresis (SDS–PAGE) gels and transferred to a polyvinylidene fluoride (PVDF) membrane. After blocking with skim milk for 1 h in Tris-buffered saline with Tween 20 (TBST), the membranes were incubated with the primary antibodies including CDKN2A (#AF5484, Affinity, 1:1,000), SDHB (#ab175225, Abcam, 1:1,000), CCS (#DF3971, Affinity, 1:1,000), ULK1 (#DF7588, affinity, 1:1,000), CMC1 (#24030-1-AP, Proteintech, 1:1,000), and ACTIN (#3700, CST, 1:1,000) at 4℃ overnight. The membranes were washed three times with TBST, 10 min each time, and subsequently incubated with horseradish peroxidase (HRP)-conjugated secondary antibody (#7074, CST, Boston, MA, USA, 1:5,000) for 1 h. Protein bands were visualized using the ECL Prime Western Blotting Detection System (32209, Thermo Fisher Scientific, Waltham, MA, USA).

### Statistical analysis

Statistical analysis was performed using R software 4.1.2 and GraphPad Prism version 8.4.1. Wilcoxon test and t-test were used to screen DEGs and others (14). We selected the prognosis genes or factors with univariate Cox regression, and LASSO Cox regression was used for construct the risk model (15). The method of how to calculate C-index could refer to this literature (16). Kaplan–Meier method was used for survival analysis (17). Quantitative values were expressed as mean ± standard deviation. p<0.05 was considered to indicate a statistically significant difference.

## Results

### The outline of this research and patients’ information

**Figure 1:**
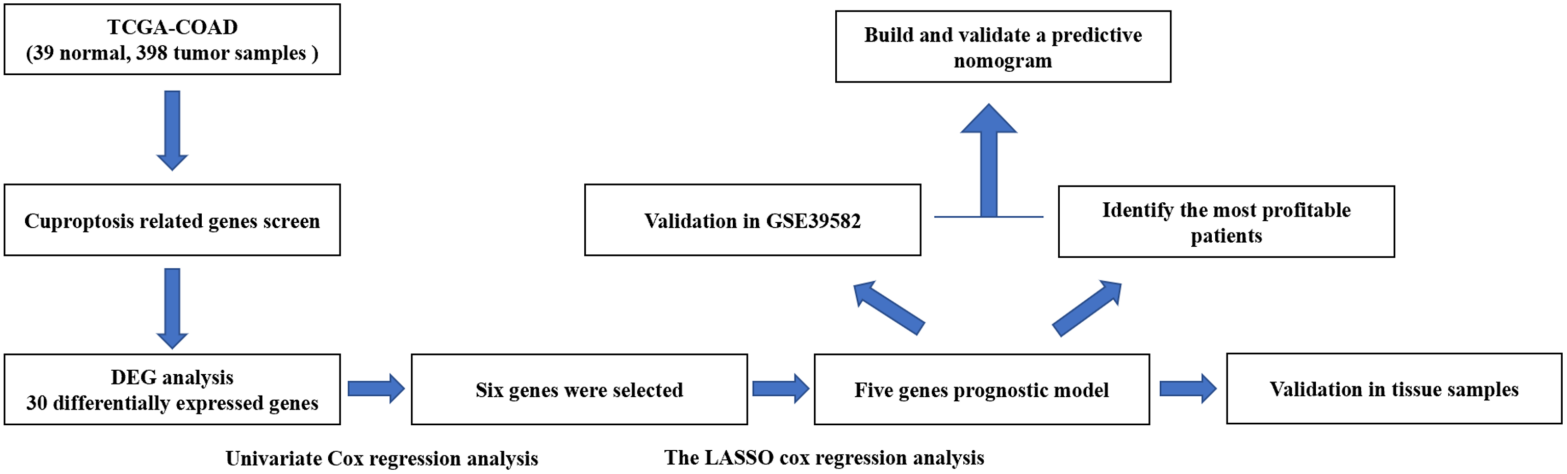
Flowchart illustrating the construction of curoptosis-related risk model in COAD patients.

The flow chart of this research is shown in **Figure 1**. The relevant clinicopathological characteristics of patients from TCGA and GEO (GSE39582) cohorts is shown in **Table 2**.

**Table 2:** Clinical information of COAD patients from TCGA and GEO.

		TCGA	GEO
		n=384	n=579
Age	≤65	159	227
	>65	225	351
	Unknown	0	1
Gender	Female	180	260
	Male	204	319
Stage	Stage 0	0	4
	Stage I–II	216	306
	Stage III–IV	157	269
	Unknown	11	0
T	T0	0	1
	T1–2	77	60
	T3–4	306	495
	Tis	1	3
M	Unknown	0	20
	M0	285	496
	M1	54	61
	Mx	39	2
	Unknown	6	20
N	N0	230	311
	N1–2	154	242
	Unknown	0	26

### Identification of differentially expressed cuproptosis-related genes

**Figure 2:**
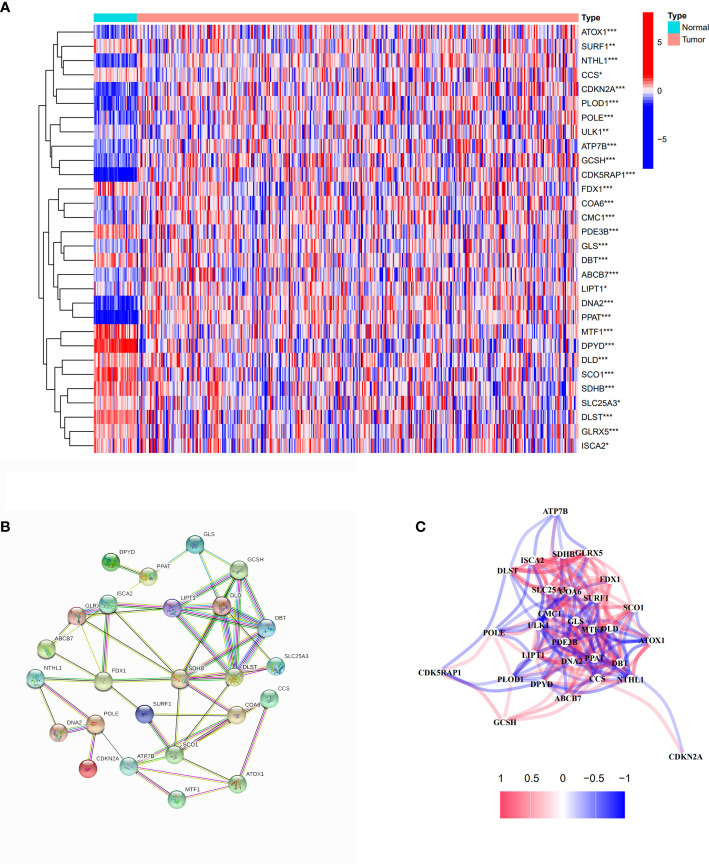
Identification of differentially expressed cuproptosis-related genes. **(A)** The heatmap of differentially expressed cuproptosis-related genes in COAD compared with normal tissues. Red spots represent upregulated genes; blue spots represent downregulated genes. *p<0.05, **p<0.01, ***p<0.001. **(B)** PPI network of differentially expressed cuproptosis-related genes. **(C)** The correlation network of differentially expressed cuproptosis-related genes.

We collected 44 cuproptosis-related genes from prior papers and extracted 30 differentially expressed genes by analyzing the expression between normal and tumor samples from TCGA cohort, which are presented in **Figure 2A**. Among them,14 genes expression was decreased (DPYD, MTF1, SCO1, PDE3B, SDHB, DLST, DBT, FDX1, DLD, GLRX5, SURF1, ISCA2, SLC25A3, and CCS), and 16 genes expression was increased (LIPT1, ULK1, ABCB7, CMC1, COA6, ATOX1, GLS, POLE, PLOD1, GCSH, ATP7B, CDK5RAP1, NTHL1, DNA2, PPAT, and CDKN2A). The PPI network result showed that FDX1, ATP7B, SCO1, SDHB, DLST, LIPT1, DLD, DBT, and GCSH were the key genes, which were associated with more other genes (**Figure 2B**). The correlation network based on 30 cuproptosis-related genes is shown in **Figure 2C**. Meanwhile, cell signaling pathways involved in DEGs were analyzed, and the results proved that differentially expressed copper-death-related genes were mainly involved in the tricarboxylic acid (TCA) cycle of cells (**Supplementary Figure S1**).

### Tumor classification based on the differentially expressed cuproptosis-related genes

**Figure 3:**
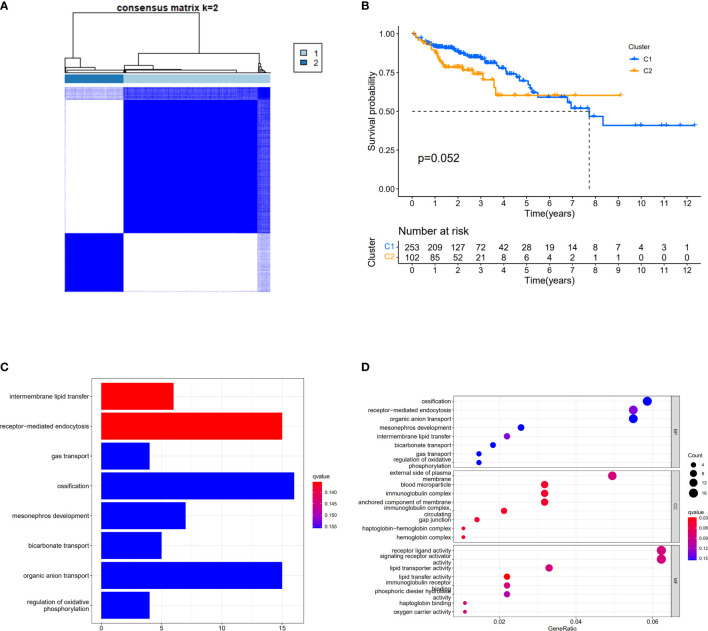
Divided COAD patients into two different clusters. **(A)** Consensus clustering matrix for k = 2. **(B)** Kaplan–Meier curves of OS for two clusters in COAD. **(C)** KEGG analysis of the curoptosis-related genes. **(D)** GO analysis of the curoptosis-related genes.

We used a consensus clustering analysis with all patients in TCGA cohort to find out the connections between the association of the 30 cuproptosis-related genes and COAD subtypes. At first, we excluded the samples whose follow-up time was no more than 30 days. We found that when the clustering variable (k)=2 (from 2 to 10), the intragroup correlations were the highest, and the intergroup correlations were the lowest, which showed that COAD patients could be well halved into two clusters based on the 30 DEGs (**Figure 3A**). The overall survival (OS) time between the two clusters appeared to have significance with p = 0.054 (**Figure 3B**). The expressions of 401 DEGs based on two different clusters and clinical characteristics between two groups were shown, and we found that there was significant difference with N stage (**Supplementary Figure S2**). The KEGG pathway enrichment analysis for 401 genes showed that these genes played roles in intermembrane lipid transfer, receptor-mediated endocytosis, and so on (**Figure 3C**). The result of GO analysis is shown in **Figure 3D**.

### Construction of a prognostic model by TCGA cohort

**Figure 4:**
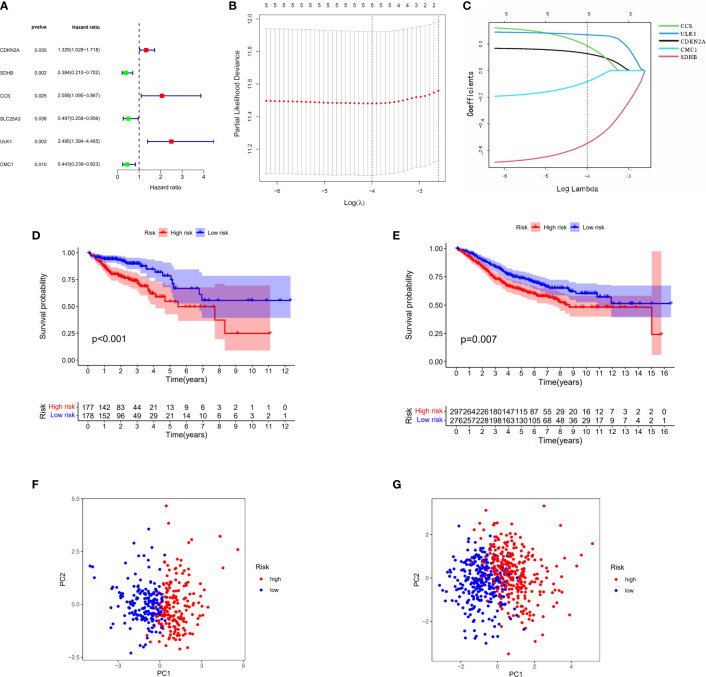
Construction of the risk score model for COAD. **(A)** Univariate Cox analysis for curoptosis-related DEGs. **(B, C)** The selection of curoptosis-related DEGs performed by LASSO regression analysis. **(D)** Survival analysis of the risk score model in TCGA. **(E)** Survival analysis of the risk score model in GEO. **(F)** PCA plot of the risk score model in TCGA. **(G)** PCA plot of the risk score model in GEO.

When the p-value was set to 0.05, six cuproptosis-related genes were selected by using univariate Cox regression analysis from 30 DEGs in TCGA cohort with 384 COAD patients. Of these genes, three (CDKN2A, CCS, and ULK1) had a positive relationship with high risk with hazard ratio (HR: the ratio of hazards of two different risk groups) >1; meanwhile, three genes (SDHB, SLC25A3, and CMC1) had a negative relationship with high risk with HRs <1 (**Figure 4A**). The table with univariate Cox regression coefficients of six genes is presented in **Supplementary Table S2**. Then, LASSO Cox regression analysis was performed to establish a prognostic risk model of five genes in the optimum λ value (0.0001) (**Figures 4B, C**). The risk score was shown as follows: risk score = (0.1274*CDKN2A) + (−0.5476*SDHB) + (0.1863*CCS) + (0.2693*ULK1) +(−0.0827*CMC1). According to the median risk score, the patients from TCGA and GEO cohorts were equally divided into two risk groups. The overall survival time of COAD patients from both two cohorts (TCGA as train set and GEO as test set) was worse in the high-risk group (**Figures 4D, E**). Furthermore, PCA indicated that patients with different risks were well divided into two clusters (**Figures 4F, G**).

### Independent prognostic value of the risk model

**Figure 5:**
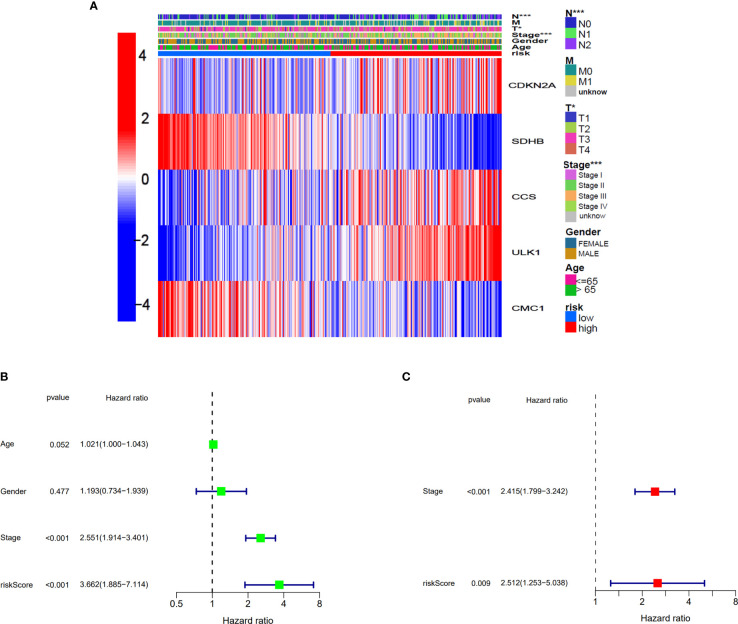
Univariate and multivariate Cox regression analyses for the risk score. **(A)** Heatmap for the connections between clinicopathological features and the risk groups. *p<0.05, ***p<0.001. **(B)** Univariate Cox analysis for the clinicopathological features and the risk score. **(C)** Multivariate Cox analysis for the clinicopathological features and the risk score.

The relationship between the expression of five genes and the two clusters of COAD patients and its corresponding clinical features in TCGA cohort is presented in **Figure 5A**. Both the univariate and multivariate Cox regression analysis indicated that the factors of stage and the risk model that we constructed have significant differences, which means that both of them were independent prognostic factors (**Figures 5B, C**).

We continued to explore which groups of patients were more compatible for our prognostic model. Patients were divided into two groups based on different clinicopathological characteristics, and then, prognostic analysis was analyzed. As presented in **Supplementary Figures S3A–F**, the patients with parameters such as age >65, male, stage I–II, T3–4, N0, and M0 seemed more compatible for our prognostic model. This result suggested that our model was more suitable for elderly COAD patients without metastasis in TCGA cohort.

### Construction of the nomogram and correlation between risk sore and clinical features

**Figure 6:**
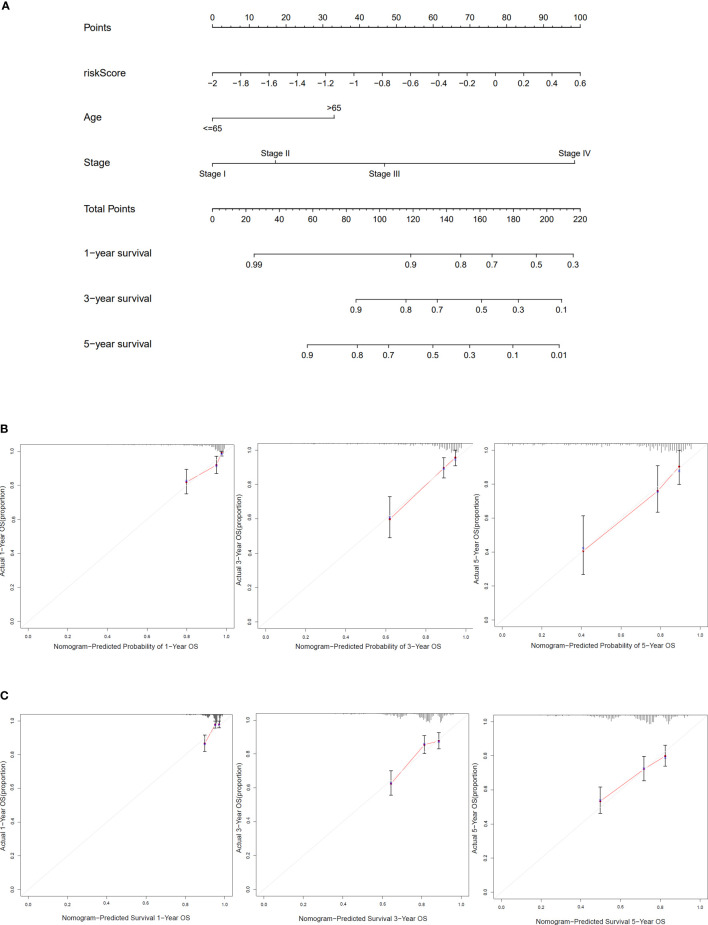
Construction and verification of the nomogram. **(A)** The nomogram based on risk score, age, and stage to predict the 1-, 3-, and 5-year survival in COAD patients. **(B)** Calibration curves for 1, 3, and 5 years of TCGA dataset. **(C)** Calibration curves for 1, 3, and 5 years of GEO dataset.

Based on clinical characteristics (age, stage, and risk score) from COAD patients in TCGA cohort, the nomogram in this study was constructed (**Figure 6A**). The C-index of the nomogram that we built was 0.785. The calibration curves of internal validation are shown in **Figure 6B**. The calibration curves of external validation are presented in **Figure 6C**. The results above implied that our risk model could well predict the prognosis of COAD patients by using this nomogram.

The risk model had a close relationship with clinical characteristics of COAD patients in TCGA cohort. We could see that the patients with stage III–IV, M1, and N1–2 were most likely to be assessed with a high risk score (**Supplementary Figures S4A–C**). The correlation between genes in the risk model and clinical features is shown in (**Supplementary Figures 5A–I**).

### Changes of T-helper cell subtypes between subgroups

**Figure 7:**
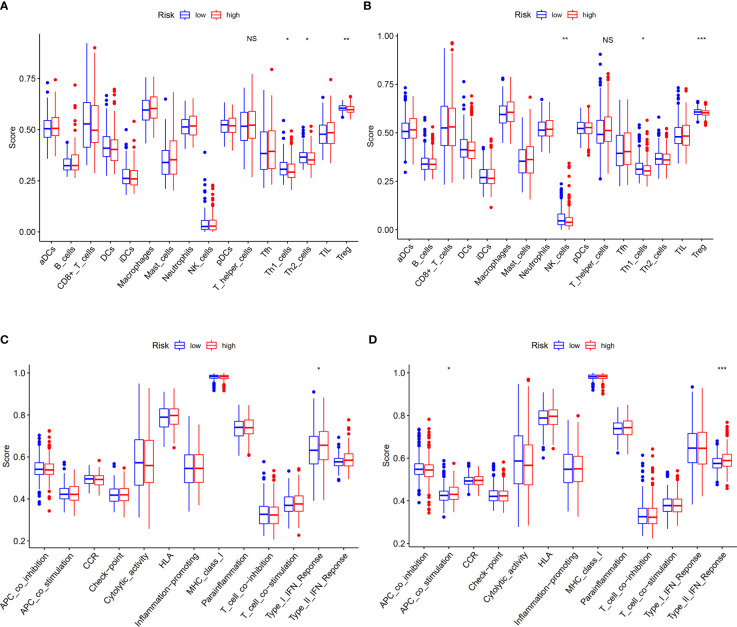
Correlation of risk score with immunological features and clinicopathological features. **(A, B)** Comparison of the enrichment scores of immune cells and immune-related pathways between low- and high-risk group in the TCGA cohort and GEO cohort. **(C, D)** Comparison of the enrichment scores of immune-related pathways between low- and high-risk group in the TCGA cohort and GEO cohort. NS, no significance. *p<0.05, **p<0.01, ***p<0.001.

Based on DEGs between the low- and high-risk groups, KEGG pathway analysis and GO enrichment analysis were performed. The results implied that the DEGs might be associated with interleukin (IL)-17 signaling pathways and humoral immune response (**Supplementary Figures S6A, B**). To explore what was different between the low- and high-risk groups with immune cells and its related function, we found that there were no differences with the level of T-helper cells (also known as CD4+T cells) (18), but its subtypes (Th1 cells and Treg cells) have significances both in TCGA and GEO cohorts (**Figures 7A, B**). When it concerned the immune-related function, we could conclude that the high-risk group of DEGs from both TCGA and GEO cohorts were mainly correlated with interferon (IFN) response (type I in TCGA and type II in GEO) (**Figures 7C, D**).

### Verification the mRNA and protein levels of genes of the risk model

**Figure 8:**
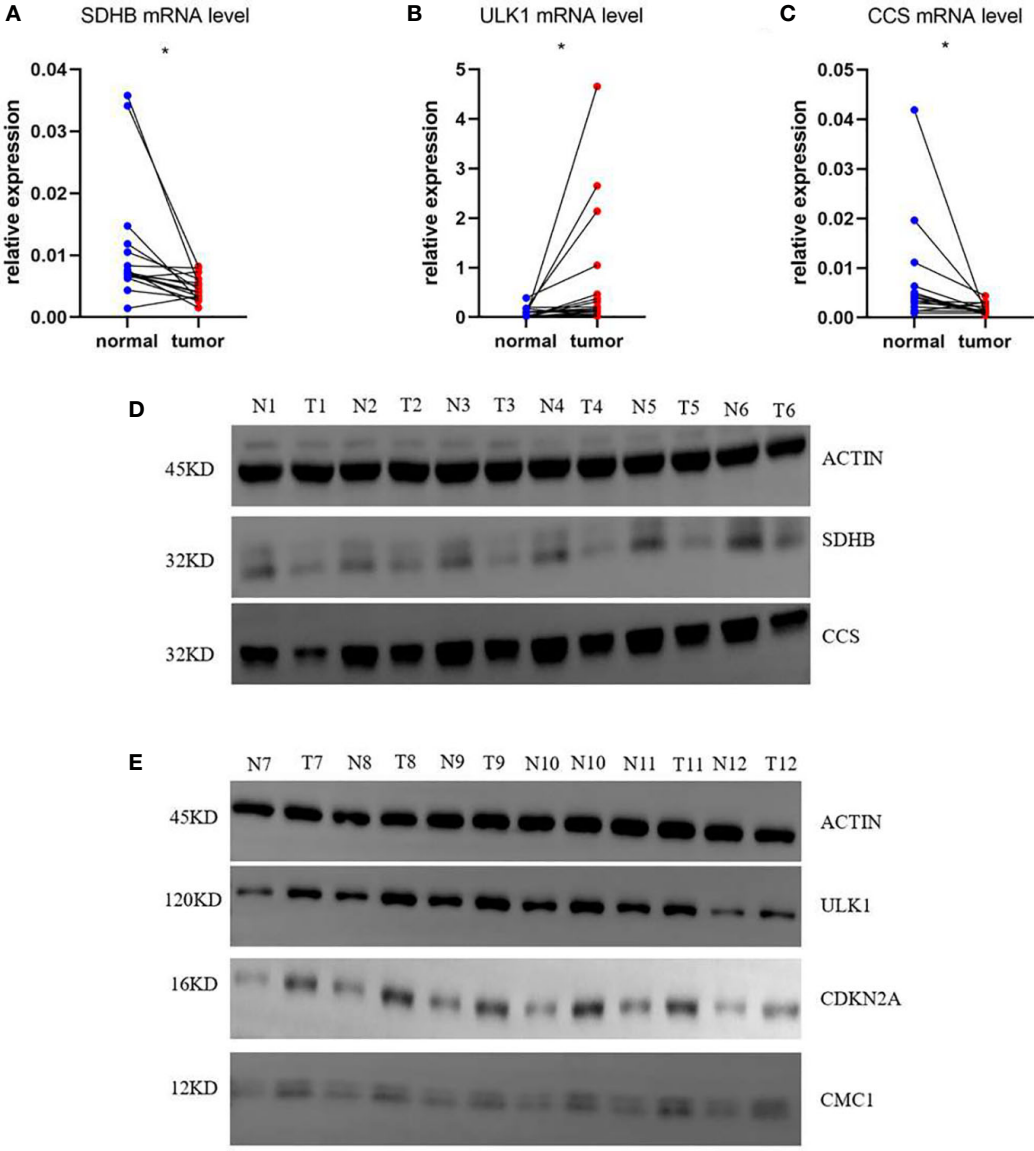
mRNA and protein relative expression of genes in the risk model. **(A)** mRNA relative expression of SDHB; **(B)** mRNA relative expression of ULK1; **(C)** mRNA relative expression of CCS; **(D)** Protein relative expressions of SDHB and CCS; **(E)** protein relative expressions of ULK1, CDKN2A, and CMC1 *p<0.05.

Based on the risk score of each gene, the weights of genes in this prognosis model from high to low should be listed by SDHB, ULK1, CCS, CDKN2A, and CMC1. Then, we collected 16 samples of COAD patients from our hospital to verify the mRNA level of five genes between normal and tumor tissues. The results showed that SDHB and CCS were decreased and ULK1 was increased with the corresponding p-values <0.05 (0.0145, 0.0498, and 0.0495) (**Figures 8A–C**). To our knowledge, the importance of the three genes in this five-gene risk model ranked top 3. The expression of CDKN2A and CMC1 did not show difference, which might require us to collect more samples for further study (**Supplementary Figures 7A, B**). Moreover, the result of Western blots showed that the expressions of SDHB and CCS were downregulated and that of ULK1, CDKN2A, and CMC1 were upregulated in tumor tissues compared with normal tissues (**Figures 8D, E**). In a word, the mRNA levels of five risk model genes were found to be broadly consistent with the DEGs in TCGA database.

## Discussion

According to global cancer statistics, colon cancer is considered as a threat to social human health and economy (1, 2). Moreover, the incidence and mortality of colon cancer are increasing with the improvement of people’s living standards, which suggests that an available prediction model of COAD patients may benefit clinical application.

As reported, cuproptosis was caused by the overload of copper ions in mitochondria, which directly combined with TCA cycle lipoylated proteins, contributing to cell death (7, 8, 19). To be more specific, a Cu-elesclomol-triggered, ferredoxin-dependent form of cell death was described as cuproptosis, which was apparently different from apoptosis, pyroptosis, necroptosis, and ferroptosis (20). In our research, we selected five cuproptosis-related genes by using TCGA and GEO databases to construct such a risk model for predicting the prognosis of patients with COAD.

According to an article of Science, loss of functions of cyclin-dependent kinase inhibitor 2A (CDKN2A) could sensitize the copper-induced cell death by elesclomol and disulfiram-diethyldithiocarbamat (two structurally distinct copper-loaded ionophores), which meant that CDKN2A might inhibit cuproptosis and be a beneficial gene for tumor cell growth (8). The hypermethylation of the promoter region of CDKN2A has been shown in many cancer types including colorectal cancer (21–24). However, the prognostic impact of CDKN2A alone in COAD has been relatively ambiguous. Succinate dehydrogenase complex iron sulfur subunit B (SDHB) belongs to Fe S cluster proteins, which are inhibited by elesclomol-induced cell death (7, 8). SDHB acts as suppressor tumor gene in colorectal cancer, and SDHB knockdown could promote tumor growth factor beta (TGFβ) signal pathway in CRC cells, which activates epithelial–mesenchymal transition (EMT) (25). Copper chaperone for superoxide dismutase (CCS) was considered to deliver copper to superoxide dismutase 1 (SOD1) and antioxidant 1 copper chaperone (ATOX1), which trafficked copper to the ATPases (ATP7A and ATP7B) (26). It was confirmed to activate the ERK1/2 signaling pathway to promote the tumor growth and migration in breast cancer (27), and there was no report about it in COAD. It was reported that copper necessarily regulated Unc-51-like autophagy-activating kinase 1 (ULK1) activity (28, 29). ULK1-mediated autophagy in colorectal cancer could be used as a target for the disulfiram/copper complex (DDC) to inhibit tumor cell growth (30). C-X9-C motif containing 1 (CMC1) was known to be involved in COX assembly, which served as a marker of copper availability (31–34). In reviews to tumor fields, few studies reported on CMC1, which is worthy of further study. Cuproptosis-related DEGs in COAD are widely related, and these genes are involved in the signaling process of TCA cycle, indicating that these five cuproptosis-related genes used to construct the risk model affect the copper death process through the TCA signaling pathway in indirect and direct ways (**Supplementary Figure S2**).

The risk model that we built based on five cuproptosis-related genes is able to predict the prognosis of COAD patients (**Figures 4D, E**) and is considered to be more suitable for elderly patients who have *in situ* invasive carcinoma (**Supplementary Figures S2A–F**). As we know, Th1 cells can produce interferon-γ, interleukin-2, and tumor necrosis factor, which activate macrophages and CD8+T cells to enhance immunity against tumor (35, 36). In our study, we found that the number of CD4+T cells did not change, but their subtypes showed differences with Th1 cells upregulated both in TCGA and GEO cohort at the high-risk group (**Figures 7A, B**). Moreover, the low-risk group tend to have enhanced expression of interferons (**Figures 7C, D**), which indicates that the mechanism of our risk model was related to the increased levels of Th1 cells to secrete more interferons against tumor. According to the results of the experimental validation of the samples, three genes (SDHB, ULK1, and CCS) had the highest risk scores and the same trend of the gene expression between tumor and normal tissues when compared with DEGs in TCGA, which means that our model has consolidated verification.

To our knowledge, this was the first time that cuproptosis-related genes had been used to construct a prognostic model in COAD and to verify gene expression of this risk model between normal and tumor tissues. Although our model could well predict the prognosis of COAD patients, there are still some shortcomings, and we need more samples and experiments to further verify the feasibility of the prognostic model and its underlying mechanisms.

## Conclusion

In summary, we first used TCGA data to screen DEGs according to cuproptosis-related genes. Next, statistical methods were used to construct prognostic risk model of patients with COAD based on DEGs. Moreover, a nomogram was built to observe clinical significance behind the risk model. Finally, the possible underlying immune mechanisms were investigated, and clinical samples were used for experimental verification.

## Data availability statement

Patients had given written informed consent for tissue collection and analysis of clinical data. The study was approved by the ethics committee of Ruijin Hospital.

## Ethics statement

The studies involving human participants were reviewed and approved by the Ethics Committee of Ruijin Hospital (Shanghai, China). The patients/participants provided their written informed consent to participate in this study.

## Author contributions

The article was written by BL, JL and AN, who contributed equally to this work. MW, XY and WC provided guidance to the manuscript preparation. All authors contributed to the article and approved the submitted version.

## Funding

This work was supported by the Shanghai Municipal Health Commission (ZK2019B17) and the Shanghai Municipal Science and Technology Commission (19441905400).

## Acknowledgment

We thank all the authors who contributed to this topic. We also thank Shanghai Institute of Immunology for providing devices and reagents.

## Conflict of interest

The authors declare that the research was conducted in the absence of any commercial or financial relationships that could be construed as a potential conflict of interest.

## Publisher’s note

All claims expressed in this article are solely those of the authors and do not necessarily represent those of their affiliated organizations, or those of the publisher, the editors and the reviewers. Any product that may be evaluated in this article, or claim that may be made by its manufacturer, is not guaranteed or endorsed by the publisher.
