# Supplementary material for: A novel defined cuproptosis-related gene signature for predicting the prognosis of colon adenocarcinoma
Source: Front Oncol. 2022 Nov 25;12:927028. doi: 10.3389/fonc.2022.927028 (PMC9732569; doi:10.3389/fonc.2022.927028)
Supplement: Supplementary file 1 [file DataSheet_1.docx]

**Supplementary Table 1.** **cuproptosis-related genes**

| FDX1 | GCSH | PLOD1 | ELP3 | ULK1 |
| --- | --- | --- | --- | --- |
| LIAS | DLST | SDHB | CDK5RAP1 | PDE3B |
| LIPT1 | DLAT | DPYD | ISCA2 | SCO1 |
| DLD | SLC31A1 | DNA2 | EFTDH | SCO2 |
| DLAT | ATP7A | NTHL1 | CTR1 | SURF1 |
| MTF1 | ATP7B | POLE | ATOX1 | COA6 |
| GLS | HSP70 | CISD1 | CCS | CMC1 |
| CDKN2A | GSH | GLRX5 | SOD1 | ABCB7 |
| DBT | ACO-2 | PPAT | SLC25A3 |  |

**Supplementary Table 2. Univariate cox regression coefficients of six genes**

| **gene** | **HR** | **HR.95L** | **HR.95H** | **pvalue** |
| --- | --- | --- | --- | --- |
| CDKN2A | 1.328843 | 1.027767 | 1.718116 | 0.03009 |
| SDHB | 0.383858 | 0.209817 | 0.702266 | 0.001891 |
| CCS | 2.057633 | 1.094887 | 3.866933 | 0.024989 |
| SLC25A3 | 0.49707 | 0.258497 | 0.955825 | 0.036136 |
| ULK1 | 2.495056 | 1.394244 | 4.465002 | 0.002075 |
| CMC1 | 0.443271 | 0.238786 | 0.822868 | 0.009947 |


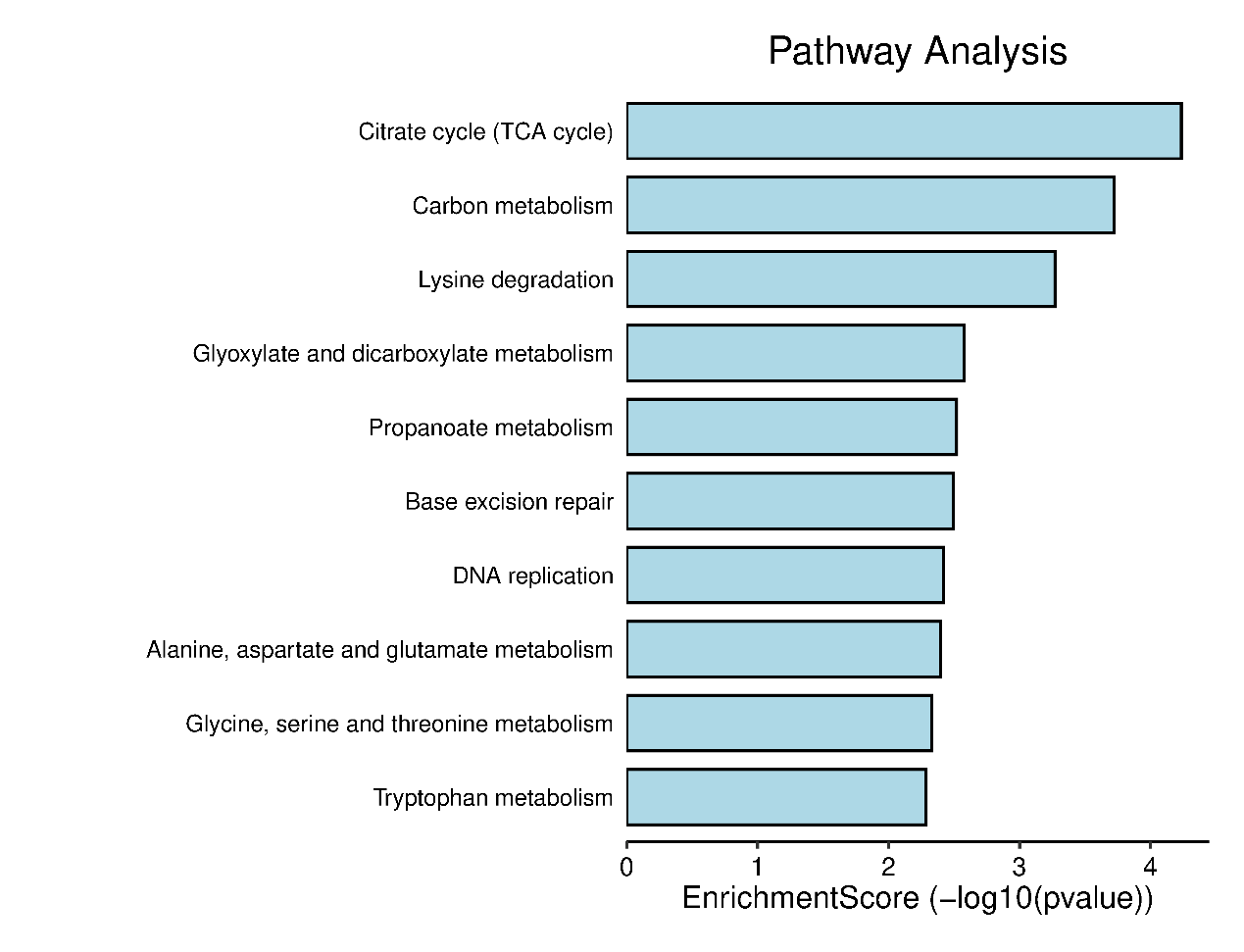


**Supplementary Figure 1.** KEGG analysis of 30 cuproptosis related DEGs.


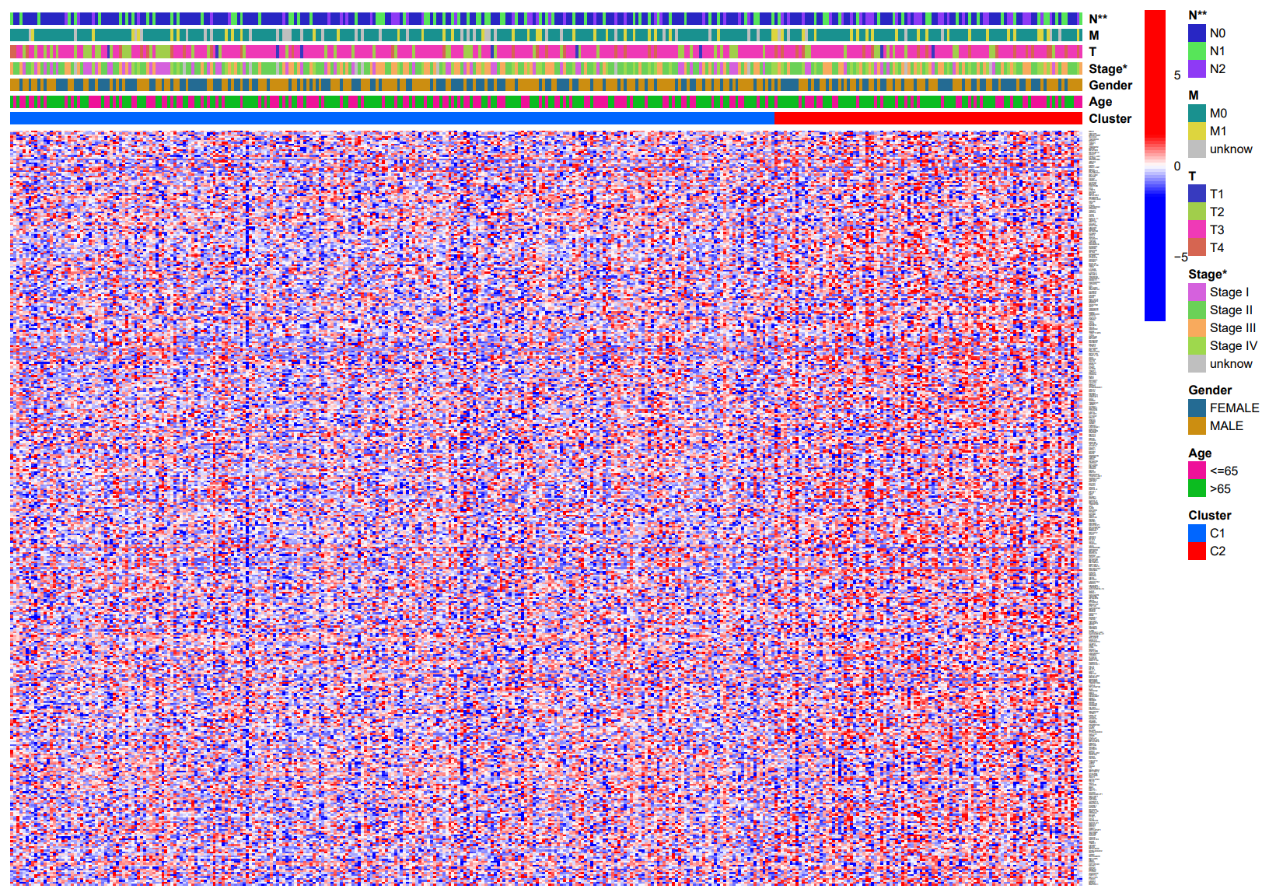


**Supplementary Figure 2.** Heatmap for curoptosis related DEGs and associated clinicopathological features between two clusters. *P<0.05, **P<0.01, ***P<0.001.


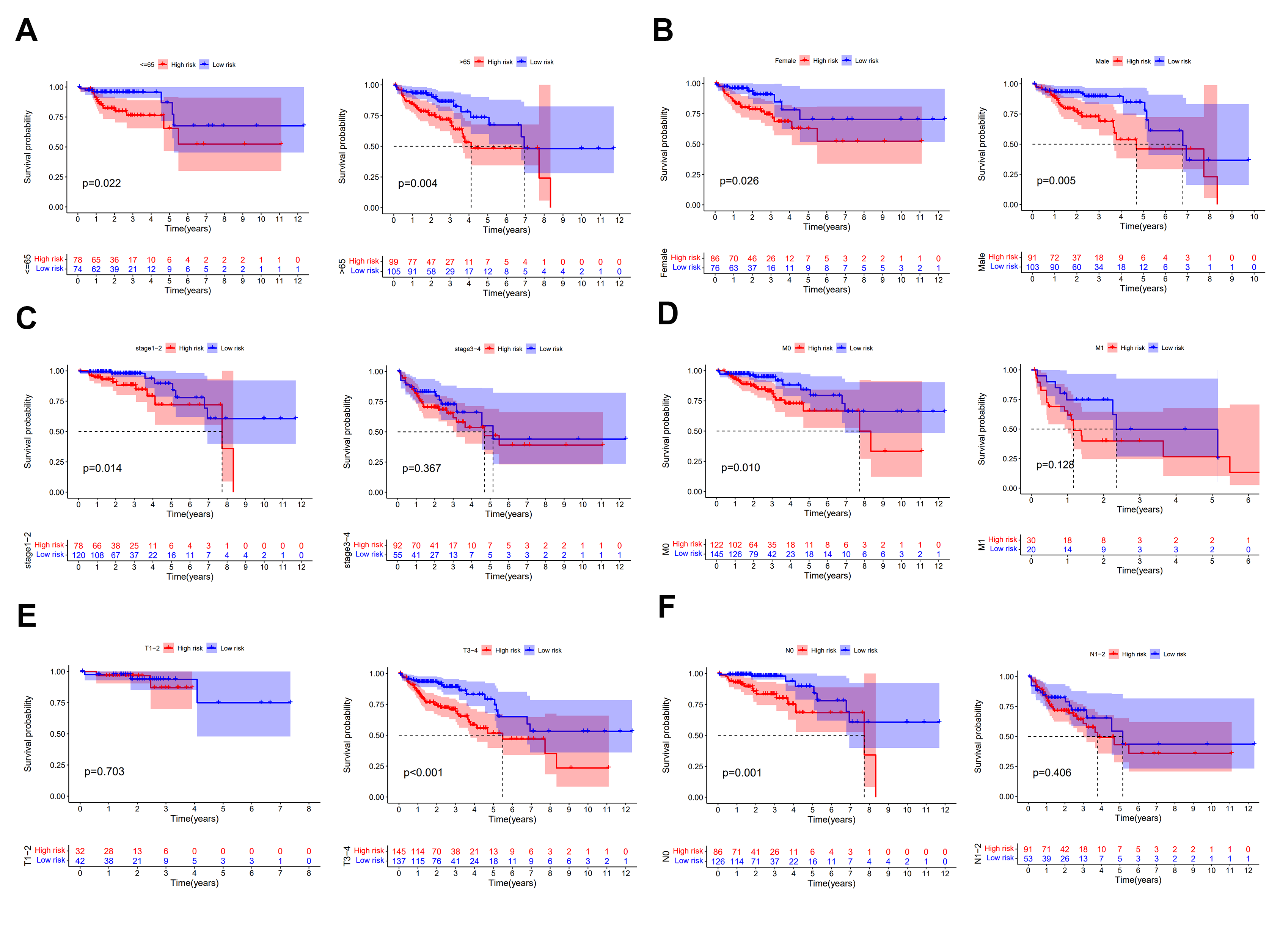


**Supplementary Figure 3. Kaplan-Meier survival curves of high- and low-risk groups among patients sorted according to different clinicopathological variables.** (A) Age (<=65;>65). (B) gender (Female; Male). (C) Stage (stage1-2; stage3-4). (D) M (M0; M1). (E) T (T1-2; T3-4). (F) N (N0; N1-2).


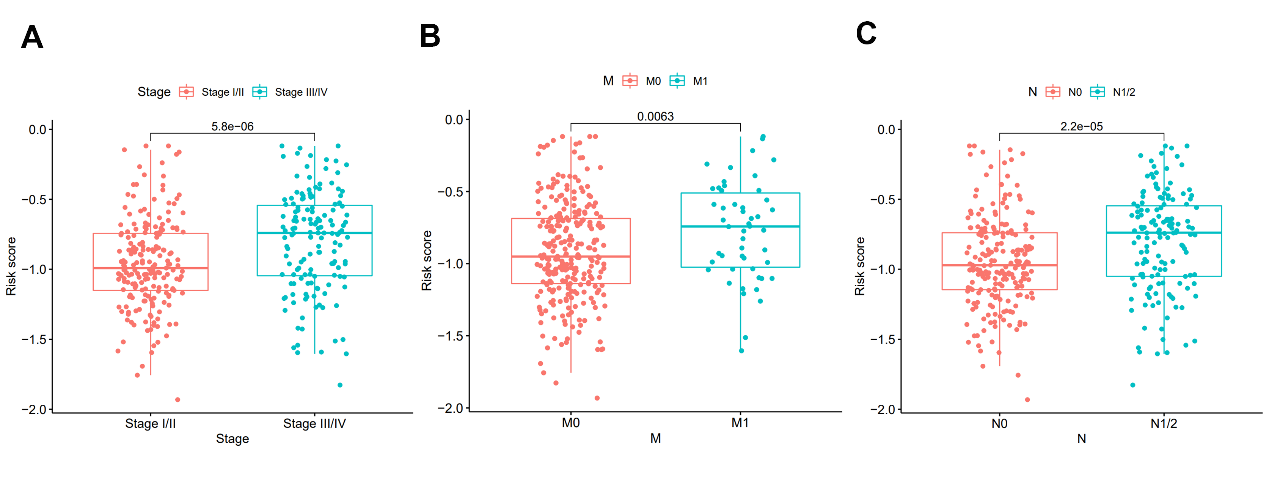


**Supplementary Figure 4.** (A-C) Correlation between the risk score and clinicopathological features (stage; M; N).


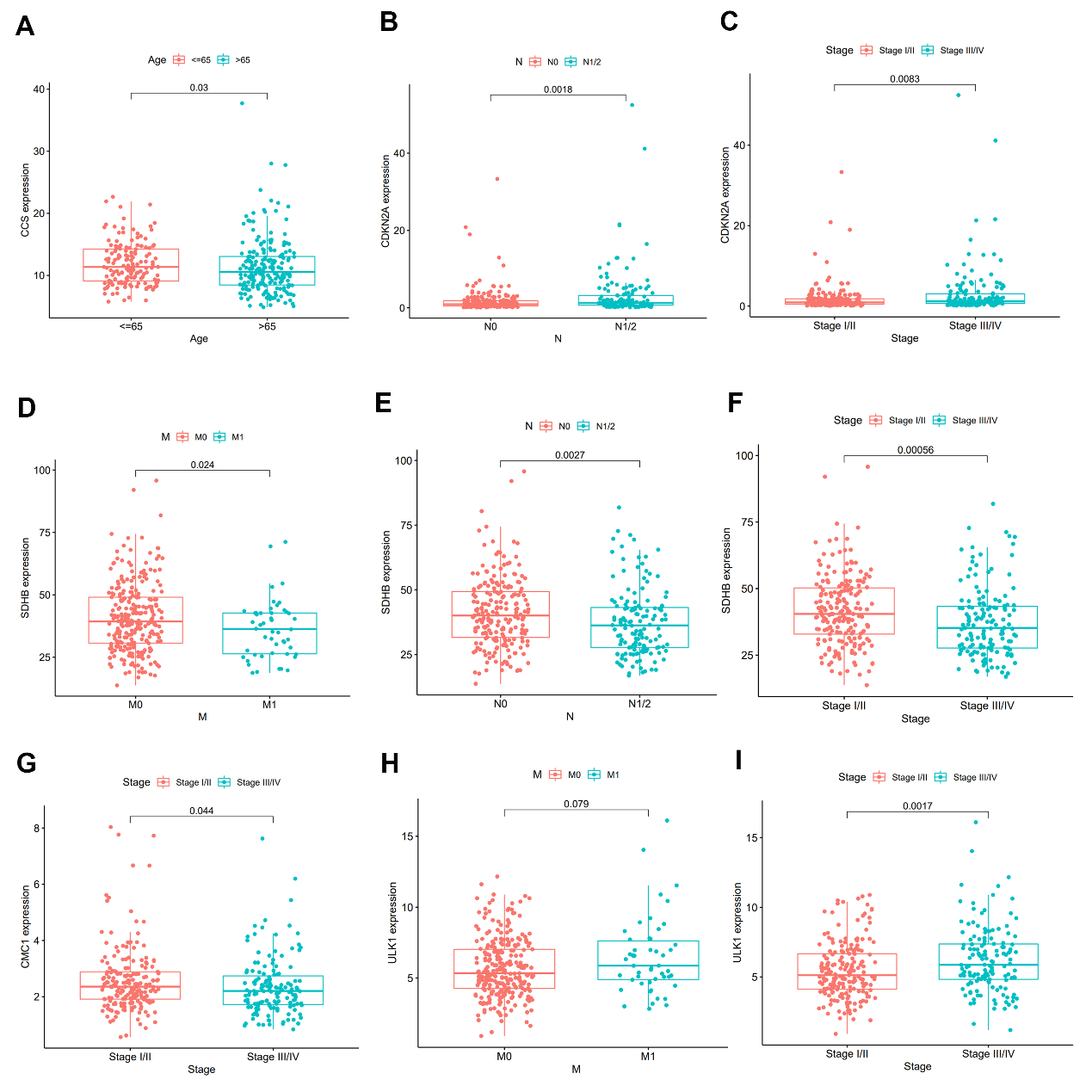


**Supplementary Figure 5. Correlation between curoptosis-related genes and clinicopathological features.** (A) Correlation of CCS with age. (B-C) Correlation of CDKN2A with N and Stage. (D-F) Correlation of SDHB with M, N and Stage. (G) Correlation of CMC1 with Stage. (H-I) Correlation of ULK1 with M and Stage.


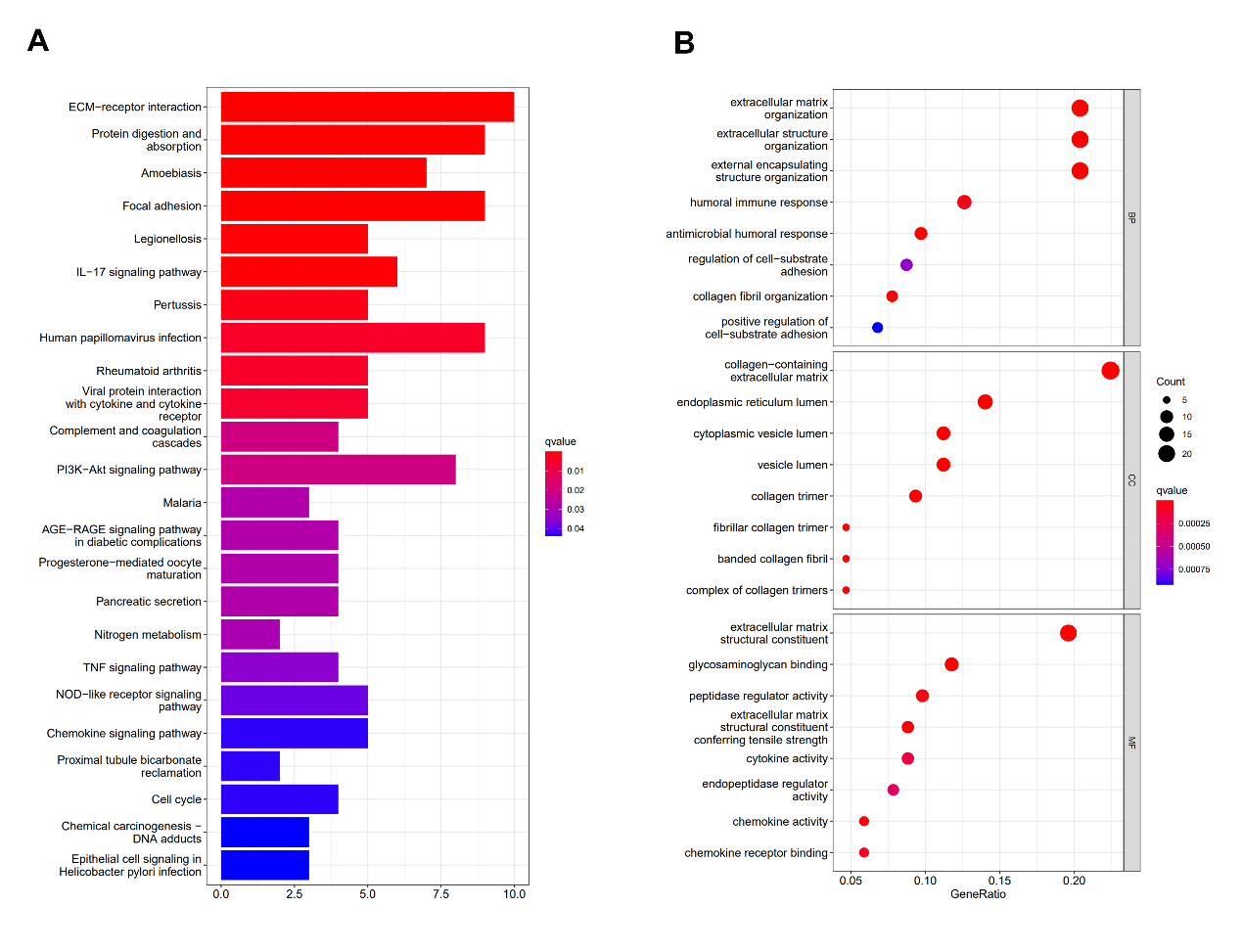


**Supplementary Figure 6. Functional analysis based on the DEGs between the two-risk groups in the TCGA cohort.** (A) Barplot graph for KEGG pathways (B) Bubble graph for GO enrichment.


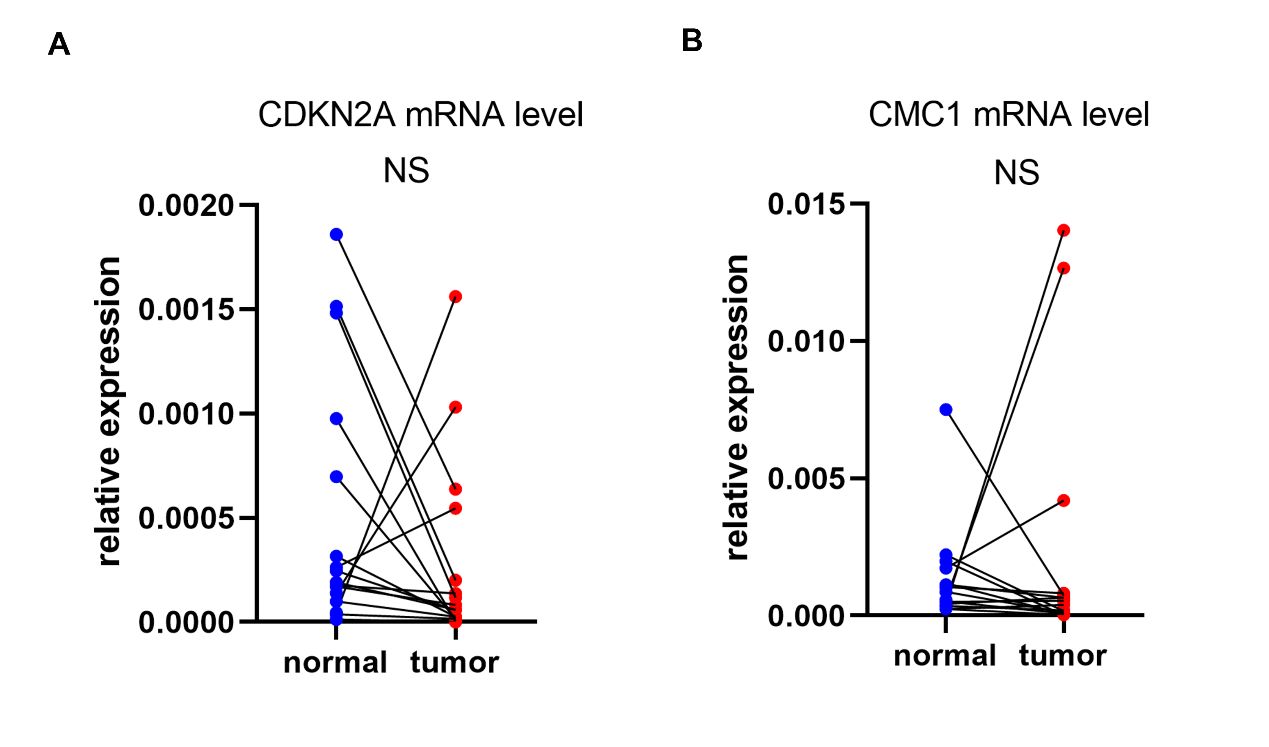


**Supplementary Figure 7. The mRNA relative expression of genes in risk model by the method of qPCR.** (A) The mRNA relative expression of CDKN2A; (B) The mRNA relative expression of CMC1.
